# Influence of History of Bronchiolitis on Health-Related Physical Fitness (Muscle Strength and Cardiorespiratory Fitness) in Children and Adolescents: A Cross-Sectional Study

**DOI:** 10.3390/muscles4010004

**Published:** 2025-02-06

**Authors:** Inês Silva de Carvalho, Rui Vilarinho, Luísa Amaral

**Affiliations:** 1 FP-I3ID, Escola Superior de Saúde Fernando Pessoa, Rua Delfim Maia 334, 4200-256 Porto, Portugal; inesilvac18@gmail.com (I.S.d.C.); lamaral@ufp.edu.pt (L.A.); 2 Centro de Investigação em Reabilitação (CIR), Escola Superior de Saúde, Instituto Politécnico do Porto, 4200-072 Porto, Portugal

**Keywords:** pediatrics, cardiorespiratory fitness, muscle strength, bronchiolitis, quality of life

## Abstract

A history of bronchiolitis may lead to changes in health-related physical fitness in children and adolescents. The aim of this study was to analyze the influence of history of bronchiolitis on health-related physical fitness components (muscle strength and cardiorespiratory fitness) in children and adolescents. A cross-sectional study was conducted, and 86 participants (aged 7–14 years; all males) were divided into two groups: a group with (n = 40) and without (n = 46) history of bronchiolitis. Muscle strength was assessed with handgrip and isometric quadriceps strength tests with handheld dynamometers and the five-times sit-to-stand test. Cardiorespiratory fitness was assessed with the six-minute walk test (6MWT). The Mann–Whitney test was used to compare the health-related physical fitness tests between groups and the binary logistic regression model was used to estimate the potential risk or protective factors in participants. No significant differences were observed between the groups in muscle strength tests. Participants without bronchiolitis walked a greater distance in the 6MWT than those with bronchiolitis (*p* = 0.002), and this appears to reproduce a protective factor in the group without bronchiolitis with an odds ratio of 0.99 [95% confidence interval: 0.98–0.99]. In this study, a history of bronchiolitis appears to influence cardiorespiratory fitness, but not muscle strength, in children and adolescents.

## 1. Introduction

Bronchiolitis, an acute respiratory viral infection of the lower airways, is the most common respiratory disease in infants and young children caused by respiratory syncytial virus (RSV). RSV is spread by respiratory droplets, either directly from an infected person or by touching contaminated surfaces and then transferring the virus to the nose, mouth, or eyes. Children with RSV bronchiolitis typically present first with upper respiratory symptoms such as fever, runny nose, and nasal congestion that last for two to four days. These symptoms are often followed by lower respiratory symptoms, including a worsening cough, wheezing, and signs of difficulty breathing [1,2].

While much attention has been devoted to understanding the acute management and short-term outcomes of bronchiolitis [3,4,5], there is a growing recognition of the need to explore its potential long-term consequences [6,7]. Early-life respiratory infections, such as bronchiolitis, have been linked to an increased risk of respiratory symptoms and reduced lung function in later childhood [8,9] and also adulthood [10]. Bronchiolitis is associated with chronic airway inflammation and remodeling, which can lead to reduced lung function, particularly in terms of airway responsiveness and airflow limitation. Studies suggest that children who have had bronchiolitis as infants are at higher risk of developing asthma-like symptoms, reduced exercise tolerance, and impaired functional capacity [11]. These problems may persist in adolescence, affecting physical activity and quality of life. In addition, repeated respiratory infections early in life can worsen these outcomes and increase the likelihood of chronic respiratory disease [12]. However, the extent to which bronchiolitis influences other health-related outcomes at earlier ages, such as physical fitness, is less well understood.

Health-related physical fitness encompasses a range of physiological and performance parameters, including cardiorespiratory fitness and muscular strength, that play a critical role in promoting the overall health and well-being of children and adolescents, and exert a central influence on their overall physical, mental, and social development [13]. Cardiorespiratory fitness refers to the body’s ability to sustain prolonged periods of moderate to vigorous physical activity that involves large muscle groups [14]. The ability to perform at this level of exertion depends on how well the respiratory, cardiovascular, and musculoskeletal systems work together. Cardiorespiratory fitness is an important aspect of physical fitness because (1) low levels of cardiorespiratory fitness are associated with a significantly higher risk of early death from all causes, particularly cardiovascular disease; (2) improvements in cardiorespiratory fitness are associated with a lower risk of death from all causes; and (3) higher levels of cardiorespiratory fitness are typically associated with higher levels of regular physical activity, which has numerous health benefits [13]. On the other hand, muscular strength is defined as the ability to exert force on an external object or resistance [15]. Strength can be evaluated either statically (without visible movement at a joint or joints) or dynamically (involving movement where the muscle changes length while moving an external load or body part). Static or isometric strength can be easily measured using tools like dynamometers [13].

Therefore, understanding the long-term effects of a history of bronchiolitis on components of physical fitness in later childhood and adolescence is critical for both clinical practice and public health policy. Research on the long-term effects of bronchiolitis is important for public health policy, as it can provide insight into the potential burden of chronic respiratory or fitness-related problems in populations. Understanding these effects helps policymakers design early-intervention programs, allocate healthcare resources efficiently, and promote preventive measures. It can also support evidence-based guidelines to improve child health outcomes and reduce future healthcare costs [16].

From a clinical perspective, recognizing the long-term effects of bronchiolitis on physical fitness is important for early detection and intervention. Clinicians can monitor at-risk individuals for signs of delayed development of physical abilities, allowing for timely interventions, such as tailored physical therapy or exercise programs, to mitigate these effects. This proactive approach can help improve the quality of life for these children by ensuring that their physical health does not interfere with their daily functioning or social participation. Beyond the individual level, the societal impact of bronchiolitis is significant. Reduced exercise capacity in individuals with a history of bronchiolitis may lead to increased use of healthcare resources due to higher rates of respiratory infections, asthma exacerbations, or long-term complications such as chronic lung disease. This, in turn, places a strain on healthcare systems and increases the economic burden on society. In addition, individuals with reduced physical fitness may experience lower levels of social participation and mental well-being, further impacting societal health outcomes [17].

By further elucidating the relationship between bronchiolitis history and physical fitness, healthcare professionals and policymakers can develop targeted, evidence-based interventions that not only improve individual health outcomes but also contribute to improved public health strategies and resource allocation.

Therefore, understanding the influence of a history of bronchiolitis on the various components of physical fitness assessment in children and adolescents is of particular importance because of its potential to affect individuals across the lifespan. Thus, this study aims to analyze the influence of the history of bronchiolitis on the components of health-related physical fitness, cardiorespiratory fitness, and muscle strength in children and adolescents.

## 2. Materials and Methods

### 2.1. Study Design and Participants

A cross-sectional study was conducted between January and May 2023. Reporting follows STrengthening The Reporting of OBservational Studies in Epidemiology (STROBE) guidelines [18], and was approved by the Ethics Committee of Fernando Pessoa University (protocol code: ESS/FSA—333/22-2).

The recruitment of participants and data collection were carried out at a soccer academy (Escola de Futebol Dragon Force) in Porto, Portugal. During a meeting with their respective parents or legal guardians, participants were informed of the existence and nature of this research project. After the initial introduction to the study, a detailed informed consent document was provided to ensure that participants and their parents or legal guardians fully understood the nature of the research. The consent form outlined the objectives of the study, the conditions of participation, and the potential risks and benefits. It also emphasized the confidentiality measures in place and provided clear instructions on how participants could contact the researchers with questions or concerns throughout the study. After reviewing the information, both participants and their parents or guardians voluntarily signed the informed consent form, indicating their agreement to participate.

Participants were considered eligible if they met the following criteria: (1) aged between 7 and 18 years and (2) could confirm whether or not they had a previous history of bronchiolitis. We chose this specific age range to ensure that participants could fully engage and effectively collaborate in completing the physical fitness tests. Participants were excluded if they had (1) acute (within the past 4 weeks) or chronic respiratory disease; (2) presence of a significant cardiac, musculoskeletal, or neuromuscular disease; (3) presence of injury/symptoms currently or in the past 6 months; (4) signs of cognitive impairment; or (5) pre-term birth.

### 2.2. Data Collection

Participants who agreed to participate in the study were contacted by the research team to schedule two assessment sessions. These sessions were scheduled to take place within 3 to 7 days of each other to allow adequate time for the application of the field tests.

At the first visit, sociodemographic, clinical, and anthropometric data were collected, and muscle strength (five-times sit-to-stand test [5TSTS], handgrip strength, and isometric quadriceps strength) was assessed. A 6MWT was also performed to assess cardiorespiratory fitness. At the second visit, another 6MWT was performed.

Sociodemographic, clinical, and anthropometric data were collected through a detailed questionnaire administered to participants and their parents or legal guardians. Sociodemographic data included information on the participants’ age and sex. Clinical data encompassed the participant’s medical history, particularly focusing on the history and severity of bronchiolitis. Anthropometric measurements such as height, weight, and body mass index (BMI) were also recorded. Additionally, the questionnaire inquired about the weekly duration of physical activity, aiming to assess the participants’ engagement in physical exercise.

### 2.3. Five-Times Sit-to-Stand Test (5TSTS)

The 5TSTS was performed in a chair (standard height 45 cm) with arms crossed over the chest and feet flat on the floor. However, for shorter participants who could not adequately support their feet on the floor, a platform raised 3 cm from the floor was used to ensure uniform test conditions for all participants. For this study, we followed the recommendations of Furlanetto et al. (2019) to conduct the test [19]. Participants were asked to stand up fully and sit down firmly as quickly as possible. To reach the full standing position, the knees had to be fully extended, whereas to reach the full sitting position, the buttocks had to be fully in contact with the chair (the participants’ backs did not have to be in contact with the chair back). Assisted use of the arms was not allowed during the trials. Participants were instructed to stand up and sit down 5 times as quickly as possible. The stopwatch was started on the command “go” and stopped at the end of the fifth full stand [19]. Participants performed three repetitions of the 5TSTS, with a 5 min rest period between the attempts, and the best performance (i.e., shortest time) was used for analysis.

### 2.4. Muscle Strenght

To measure handgrip strength, we used a handgrip dynamometer (W50174, Baseline, Nailsea, UK). Handgrip strength is a widely recognized measure of muscular strength, particularly reflecting the force generated by the muscles in the forearm. It serves not only as an indicator of strength in the upper body but also provides insights into overall physical fitness and health. Research has shown that handgrip strength is correlated with various aspects of general health, including mobility, muscle mass, and even cardiovascular health, making it a valuable tool in both clinical and research settings [20]. Handgrip strength is typically assessed using a handheld dynamometer, a device designed to measure the maximum force exerted by the participant’s grip. To ensure consistency and accuracy, participants were asked to sit in a standardized position during the test. This position required the participant to sit with the shoulders adducted (arms close to the body), elbows flexed at a 90-degree angle, and forearms in a neutral position, with the wrist straight and the hand comfortably positioned around the dynamometer handle. The dominant hand was selected for the test in order to obtain the highest possible measure of strength. Participants are instructed to squeeze the dynamometer handle as forcefully as possible. To ensure that the results are not influenced by fatigue or technique, three separate measurements are taken, with a 1 min rest period between each attempt. This rest period helps reduce the impact of muscle fatigue, allowing the participant to produce their maximum effort during each measurement. The highest value obtained from these three attempts is typically recorded and used for analysis, as it best represents the participant’s peak handgrip strength. [21].

Quadriceps isometric strength in the dominant leg was measured with a handheld dynamometer (microFET2, Hoggan Health, The best Salt Lake City, UT, USA). The dynamometer was positioned and securely fixed horizontally on the anterior region of the participant’s leg, using a non-extensible strap at the point of force application, which was 5 cm above the lateral malleolus. During the assessment, the participants remained seated with their feet off the ground, preferably without lumbar spine support, and maintained a slight thoracic kyphosis to avoid pelvic movement. The upper limbs were crossed over the chest throughout the assessment. The measurement was conducted with the knee and hip flexed at 90 degrees, requiring the use of a wedge placed behind the thigh, with its densest part at the popliteal region to ensure the horizontal alignment of the femoral segment. For the measurement itself, specific instructions were developed and given to the participants:

*“The goal of this test is to measure your maximum strength. The movement required is an extension of the leg relative to the thigh, that is, straightening your leg. You must exert as much force as possible. I repeat, it is very important to exert maximum force. I will count down to the moment when you push against the strap to perform the movement, and I will encourage you for a few seconds. After that, you can relax and stop. During the test, I will ask you to keep your arms crossed over your chest and slightly bend your back. Please avoid fully bending or straightening your back during the measurement. We will repeat the test several times. This is a maximum effort test”.*

Verbal encouragement was vigorous and delivered with consistent intensity throughout all measurements, using phrases like “Go, go, go!” or “Push, push, push!”. The contraction lasted for at least 4 s with a maximum of 6 s, with a recovery period of 30 to 60 s between repetitions. The first two measurements were excluded, as they served as a warm-up and familiarization phase, allowing for the assessment of measurement stability. Subsequently, the best of three acceptable and reproducible attempts (defined by variations of less than 10% between the two highest values) were recorded. During each repetition, the participant performed an inhalation followed by an exhalation while simultaneously executing maximum quadriceps contraction according to the evaluator’s instructions. The maximum value obtained had been recorded in kilogram-force (KgF) [22].

### 2.5. Six-Minute Walk Test

Cardiorespiratory fitness was measured by distance walked on the 6MWT, in a 30 m corridor. This is a self-paced test of walking capacity and participants were asked to walk as far as possible in 6 min along a flat corridor. Standardized instructions and encouragement were given during the test according to the American Thoracic Society (ATS) guidelines [23]. This test requires a flat, hard-surfaced, straight corridor at least 30 m long to ensure consistency, and the area should be free of traffic to avoid interruptions and distractions. Safety precautions were ensured and essential equipment was used, including resting chairs, a stopwatch, a pulse oximeter, a sphygmomanometer, the standardized Borg perceived exertion scale [24], and a pre-marked course.

Participants wore comfortable clothing and footwear, and refrained from vigorous exercise two hours prior to the test. Before the test, baseline measurements, including resting heart rate, blood pressure, oxygen saturation (SpO_2_), and perceived exertion score, were recorded. The goal of this test was for the participant to walk as far as possible within six minutes without running or jogging. During the test, the participants were monitored continuously. Standardized encouragement phrases, such as “You are doing well, keep it up,” were given at regular intervals to maintain consistency and motivation, according to ATS guidelines. After the test, the total distance walked, the number of laps completed, and any partial distances were counted. Two tests were performed, with a rest period of at least 30 min provided between tests, and the best performance (i.e., longest distance) was used in the analysis.

### 2.6. Statistical Analysis

Statistical analysis was performed using the IBM SPSS Statistics, version 27. The level of significance was set at 0.05. Continuous variables were tested for normality using the Shapiro–Wilk test. Since the data did not meet the assumption of normality, descriptive statistics were presented using the median and percentiles.

Differences in muscle strength tests and cardiorespiratory fitness between the groups (with or without bronchiolitis) were calculated with the Mann–Whitney (U) test.

The binary logistic regression model, reporting the respective adjusted odds ratios and confidence intervals, and adjusted for all co-variables (handgrip strength, quadriceps isometric muscle strength, 5TSTS, and 6MWT), was applied with the aim of estimating potential risk or protection factors in participants with or without a history of bronchiolitis.

## 3. Results

**Figure 1:**
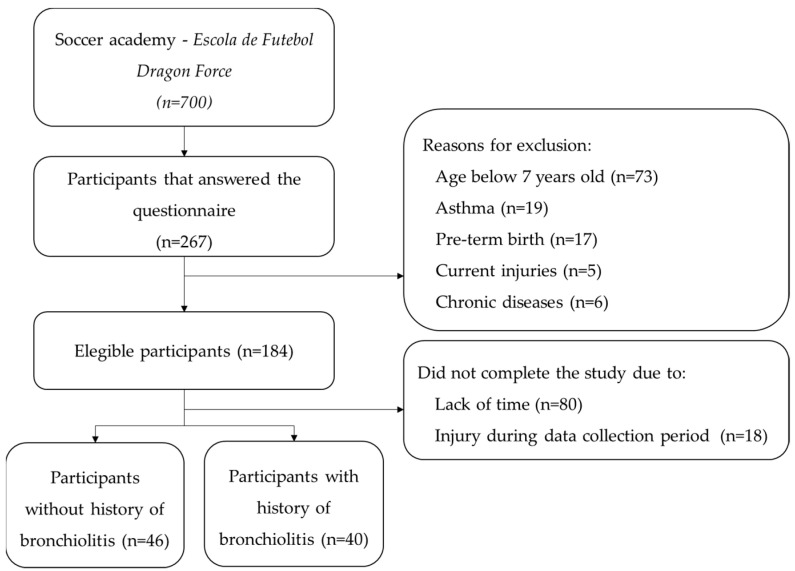
Flow chart of patients through the study.

Two hundred and sixty-seven participants agreed to participate and answered the questionnaire; however, after applying the eligibility criteria and removing participants who dropped out, eighty-six participants completed the study (Figure 1).

These participants were divided into two groups: one group with 40 participants with a history of bronchiolitis, and another group with 46 participants without a history of bronchiolitis. The characteristics of the 86 participants enrolled in the study, overall and by group, are shown in Table 1. No differences were found in participants’ characteristics between the groups (Table 1).

In the group with a history of bronchiolitis, according to bronchiolitis severity, 28 participants were classified with mild, 4 participants with moderate, and 8 participants with severe bronchiolitis.

### Muscle Strength and Cardiorespiratory Fitness Comparisons Between Groups

No significant differences were observed in the muscle strength tests (5TSTS, handgrip strength test, and quadriceps isometric muscle strength) between the groups (0.665 < *p* < 0.927) (Table 2). In terms of the cardiorespiratory fitness test, a significant difference (*p* = 0.002) was found between the groups, with the group without bronchiolitis performing the longest distance (Table 2).

Table 3 shows the binary logistic regression adjusted for all co-variables, where only the cardiorespiratory variable (best distance performed in the 6MWT) was significantly associated with having history of bronchiolitis, with an odds ratio of 0.99 [95% CI: 0.98–0.99] (Table 3).

## 4. Discussion

The present study aimed to explore the influence of history of bronchiolitis on components of health-related physical fitness, specifically muscle strength and cardiorespiratory fitness, in children and adolescents. Our findings provide valuable insights into the long-term impact of bronchiolitis on these fitness components. However, to the best of our knowledge, no studies have specifically examined the influence of the history of bronchiolitis on health-related physical fitness components in pediatric populations, making direct comparisons with existing research challenging.

Our study included 86 participants, divided into two groups based on their history of bronchiolitis: 40 participants with a history of bronchiolitis and 46 without. Participant characteristics were similar between the two groups, indicating a well-matched cohort for comparison.

Interestingly, our analysis did not reveal significant differences in muscle strength between the groups, as measured by the 5TSTS, handgrip strength test, and quadriceps isometric strength, indicating a lack of association between bronchiolitis history and muscle strength in our study. This finding suggests that bronchiolitis in early childhood does not have a discernible long-term effect on muscle strength in children and adolescents. The underlying mechanisms may involve the rapid muscle growth and development that exists at this stage of life or the possibility that muscle strength is more influenced by genetic, nutritional, and physical activity-related factors over time [25] than by early respiratory disease. Additionally, the sample for this study was recruited from a soccer academy, which may suggest that the participants have higher baseline levels of physical activity and muscle strength due to their athletic training [26]. This could potentially mitigate the long-term impact of early respiratory illness on muscle function.

However, a significant difference was observed in cardiorespiratory fitness, as assessed by the 6MWT. Participants without a history of bronchiolitis performed better, covering a significantly longer distance compared to those with a history of bronchiolitis. This finding aligns with the existing literature, suggesting that early-life respiratory illnesses can have long-term consequences on pulmonary function and overall aerobic capacity [9,27]. The reduced cardiorespiratory fitness observed in the bronchiolitis group may be due to altered lung growth, potentially impairing aerobic performance. However, this hypothesis should be confirmed through pulmonary function assessments.

The binary logistic regression analysis further underscored the importance of cardiorespiratory fitness as the best distance performed in the 6MWT was significantly associated with a history of bronchiolitis, with an odds ratio of 0.99 [95% CI: 0.98–0.99]. Bronchiolitis can lead to persistent airway inflammation, reduced lung function, and increased airway reactivity that may extend into later childhood [11]. Reduced cardiorespiratory performance among individuals with a history of bronchiolitis may reflect underlying respiratory limitations, as well as adaptations such as reduced habitual physical activity due to past breathing difficulties or reduced exercise tolerance. These results align with prior studies showing that respiratory infections in early childhood can have lasting impacts on pulmonary function and, by extension, physical fitness [28]. Thus, these findings underscore the importance of monitoring cardiorespiratory fitness in children with a history of bronchiolitis, as well as developing targeted interventions that encourage physical activity and improve lung health. Further longitudinal studies could clarify whether interventions aimed at improving fitness in children with a history of bronchiolitis can mitigate some of the observed deficits in physical fitness, potentially improving health outcomes across their lifespan [29].

The results of this study highlight several important implications for clinical practice and future research regarding the long-term effects of bronchiolitis on children’s physical health, particularly regarding cardiorespiratory fitness and muscle strength. Given the association between a history of bronchiolitis and reduced aerobic capacity, it is critical to prioritize regular monitoring of cardiorespiratory fitness in children who have experienced this respiratory illness. Early identification of those at risk for reduced aerobic capacity may lead to timely interventions, potentially preventing further decline in physical health as these children grow older.

One promising approach to mitigating the long-term effects of bronchiolitis on physical fitness is the implementation of tailored physical activity programs. Such programs could be designed to meet the specific needs of children with a history of bronchiolitis, focusing on improving cardiorespiratory fitness and strengthening muscle function. Research has shown that physical activity interventions can improve aerobic capacity and muscle strength in populations with respiratory disease [30,31]. Therefore, introducing these interventions early in the recovery process could significantly reduce the risk of permanent health problems. By incorporating strategies such as aerobic exercise and strength training, healthcare providers can address the unique challenges these children face in maintaining a healthy level of physical fitness.

In addition to these immediate interventions, further investigation of the long-term course of lung function, muscle strength, and cardiorespiratory fitness in children who have had bronchiolitis is essential. Longitudinal studies are needed to follow these health measures over time and to determine the trajectory of physical fitness in this population. Such studies could provide valuable insights into the persistence of cardiorespiratory impairments and the factors that influence recovery or further decline. In addition, the results of these studies could inform the development of more effective rehabilitation strategies tailored to the needs of children who have experienced bronchiolitis.

Another critical aspect that future research should address is the stratification of patients based on the severity of their bronchiolitis and lung function assessment. Understanding how the severity of the disease affects long-term physical health could be crucial in tailoring interventions more effectively. It is likely that children with more severe cases of bronchiolitis are at greater risk of permanent impairment of cardiorespiratory fitness and lung function. Therefore, severity-based stratification would allow for more targeted approaches to managing the health of these children, focusing resources on those at higher risk for physical fitness complications. Future research should also aim to account for potential confounders, such as socioeconomic status, diet, and pre-existing medical conditions, which may influence physical fitness outcomes.

Additionally, the homogeneity of the study sample (male soccer players) limits generalizability, as findings may not be representative of females, non-athletes, or other sports. Future research should include diverse populations to ensure broader applicability of conclusions regarding bronchiolitis and physical fitness outcomes.

Focusing on cardiorespiratory health, it is important to explore the mechanisms underlying the associations between bronchiolitis and long-term physical impairment. Research should aim to uncover the biological and physiological pathways by which bronchiolitis may contribute to permanent reductions in physical fitness. For example, understanding how bronchiolitis affects lung development and muscle strength could identify potential therapeutic targets to improve health outcomes in these individuals. Identifying these mechanisms will also help refine existing intervention strategies, making them more effective in addressing the underlying causes of the physical impairments observed in children with a history of bronchiolitis.

Ultimately, the goal of future research should be to develop comprehensive, evidence-based interventions that support the long-term cardiorespiratory health of individuals with a history of bronchiolitis. By combining early intervention, tailored rehabilitation programs, longitudinal follow-up, and a deeper understanding of the underlying mechanisms, healthcare providers can better support these children as they grow into healthy adults. Addressing these issues now will help prevent long-term physical limitations and improve the overall quality of life for children affected by bronchiolitis.

## 5. Conclusions

Our study suggests that while muscle strength may remain unaffected, a history of bronchiolitis is associated with reduced cardiorespiratory fitness in children and adolescents. This indicates that early respiratory illnesses, such as bronchiolitis, may have lasting effects on physical fitness, specifically the ability to perform endurance activities. The reduced cardiorespiratory fitness observed in this population may highlight the need for targeted interventions aimed at improving aerobic capacity and overall physical health in individuals with a history of bronchiolitis. These findings emphasize the importance of addressing and continuously monitoring physical fitness in this group to promote long-term health and well-being, and prevent further complications.

## Figures and Tables

**Table 1:** Characteristics of participants.

	Total Sample (n = 86)	Without Bronchiolitis (n = 46)	With Bronchiolitis (n = 40)	*p*-Value
**Age, years**	10 [8–11]	10 [8–10.25]	9.5 [8.25–11]	0.767
**Height, m**	1.37 [1.31–1.45]	1.36 [1.30–1.45]	1.39 [1.31–1.47]	0.590
**Body mass, kg**	32 [29–37]	32 [28.5–37]	32 [29–38]	0.853
**BMI, kg/m^2^**	16.87 [15.64–18.64]	17.18 [16.02–18.68]	16.79 [15.56–18.66]	0.558
**Years of football practice**	3.5 [1–5]	3 [1–5]	4 [2.5–5]	0.168
**Hours of practice, minutes/weekly**	250 [152–260]	153.5 [152–260]	260 [152–260]	0.138

The values are expressed as medians [percentiles 25–75]. *p*-value corresponds to the comparison of values between groups. BMI, body mass index.

**Table 2:** Health-related physical fitness component values and comparisons between groups.

	Without Bronchiolitis (n = 46)	With Bronchiolitis (n = 40)	*p*-Value
**Handgrip strength, kg**	16.1 [6.70–32.50]	15.70 [6.50–57.60]	0.927
**Quadriceps strength, kg**	16.7 [8.30–39.50]	16.9 [7.40–30.50]	0.665
**5TSTS, seconds**	5.28 [3.66–7.34]	5.33 [3.31–7.70]	0.866
**6MWT, m**	633.6 [420.0–810.50]	602.5 [416.40–730.0]	0.002

The values are expressed as medians [percentiles 25–75]. *p*-value corresponds to the comparison of values between groups. 5TSTS, five-times sit-to-stand test; 6MWT, six-minute walk test.

**Table 3:** Predictive factors in the group with bronchiolitis and without bronchiolitis.

	aOR	95% CI	*p*-Value
**Handgrip strength, kg**	1.07	0.99–1.16	0.100
**Quadriceps strength, kg**	0.94	0.86–1.03	0.194
**5TSTS, seconds**	0.70	0.60–1.57	0.887
**6MWT, m**	0.99	0.98–0.99	0.022

5TSTS, five-times sit-to-stand test; 6MWT, six-minute walk test; aOR, adjusted odds ratio; CI, confidence interval.

## Data Availability

The data are available upon request from the corresponding author.
